# Oral Administration of Porphyromonas gingivalis Alters the Gut Microbiome and Serum Metabolome

**DOI:** 10.1128/mSphere.00460-18

**Published:** 2018-10-17

**Authors:** Tamotsu Kato, Kyoko Yamazaki, Mayuka Nakajima, Yasuhiro Date, Jun Kikuchi, Koji Hase, Hiroshi Ohno, Kazuhisa Yamazaki

**Affiliations:** a Research Unit for Oral-Systemic Connection, Division of Oral Science for Health Promotion, Niigata University Graduate School of Medical and Dental Sciences, Niigata, Japan; b Laboratory for Intestinal Ecosystem, RIKEN Centre for Integrative Medical Sciences (IMS), Yokohama, Japan; c RIKEN Center for Sustainable Resource Science, Yokohama, Japan; d Division of Biochemistry, Faculty of Pharmacy, Keio University, Tokyo, Japan; e Intestinal Microbiota Project, Kanagawa Institute of Industrial Science and Technology, Kawasaki, Japan; University of Kentucky

**Keywords:** *Porphyromonas gingivalis*, gut microbiome, metabolism, periodontitis

## Abstract

Periodontal disease induced by periodontopathic bacteria like Porphyromonas gingivalis is demonstrated to increase the risk of metabolic, inflammatory, and autoimmune disorders. Although precise mechanisms for this connection have not been elucidated, we have proposed mechanisms by which orally administered periodontopathic bacteria might induce changes in gut microbiota composition, barrier function, and immune system, resulting in an increased risk of diseases characterized by low-grade systemic inflammation. Accumulating evidence suggests a profound effect of altered gut metabolite profiles on overall host health. Therefore, it is possible that P. gingivalis can affect these metabolites. To test this, C57BL/6 mice were administered with P. gingivalis W83 orally twice a week for 5 weeks and compared with sham-inoculated mice. The gut microbial communities were analyzed by pyrosequencing the 16S rRNA genes. Inferred metagenomic analysis was used to determine the relative abundance of KEGG pathways encoded in the gut microbiota. Serum metabolites were analyzed using nuclear magnetic resonance (NMR)-based metabolomics coupled with multivariate statistical analyses. Oral administration of P. gingivalis induced a change in gut microbiota composition. The distributions of metabolic pathways differed between the two groups, including those related to amino acid metabolism and, in particular, the genes for phenylalanine, tyrosine, and tryptophan biosynthesis. Also, alanine, glutamine, histidine, tyrosine, and phenylalanine were significantly increased in the serum of P. gingivalis-administered mice. In addition to altering immune modulation and gut barrier function, oral administration of P. gingivalis affects the host’s metabolic profile. This supports our hypothesis regarding a gut-mediated systemic pathology resulting from periodontal disease.

**IMPORTANCE** Increasing evidence suggest that alterations of the gut microbiome underlie metabolic disease pathology by modulating gut metabolite profiles. We have shown that orally administered Porphyromonas gingivalis, a representative periodontopathic bacterium, alters the gut microbiome; that may be a novel mechanism by which periodontitis increases the risk of various diseases. Given the association between periodontal disease and metabolic diseases, it is possible that P. gingivalis can affect the metabolites. Metabolite profiling analysis demonstrated that several amino acids related to a risk of developing diabetes and obesity were elevated in P. gingivalis-administered mice. Our results revealed that the increased risk of various diseases by P. gingivalis might be mediated at least in part by alteration of metabolic profiles. The findings should add new insights into potential links between periodontal disease and systemic disease for investigators in periodontal disease and also for investigators in the field of other diseases, such as metabolic diseases.

## Abstract (precis)

Increasing evidence suggest that alterations of the gut microbiome underlie metabolic disease pathology by modulating gut metabolite profiles. We have shown that orally administered Porphyromonas gingivalis, a representative periodontopathic bacterium, alters the gut microbiome; that may be a novel mechanism by which periodontitis increases the risk of various diseases. Given the association between periodontal disease and metabolic diseases, it is possible that P. gingivalis can affect the metabolites. Metabolite profiling analysis demonstrated that several amino acids related to a risk of developing diabetes and obesity were elevated in P. gingivalis-administered mice. Our results revealed that the increased risk of various diseases by P. gingivalis might be mediated at least in part by alteration of metabolic profiles. The findings should add new insights into potential links between periodontal disease and systemic disease for investigators in periodontal disease and also for investigators in the field of other diseases, such as metabolic diseases.

## INTRODUCTION

There is an increasing amount of evidence suggesting that periodontal disease is associated with an increased risk of various metabolic, inflammatory, and autoimmune diseases, such as type 2 diabetes, atherosclerotic vascular diseases, and rheumatoid arthritis (1). Periodontal disease is a chronic inflammatory condition of the periodontium, which is induced by periodontopathic bacteria, such as Porphyromonas gingivalis. This bacterium destroys tooth-supporting tissue, resulting in tooth loss if left untreated. Histological analyses reveal a disruption in the structural integrity of the gingival epithelium that results in the exposure of capillaries to periodontal pockets (2). A dense infiltration of lymphocytes, macrophages, and dendritic cells is also observed in the surrounding connective tissue, suggesting a robust immune response to these bacteria. These immune cells, epithelial cells, and fibroblasts have also been reported to produce significant quantities of diverse proinflammatory cytokines (3).

Given these pathological features, bacteremia, endotoxemia, and the invasion of locally produced proinflammatory cytokines into the systemic circulation have been considered the causal mechanisms by which periodontal disease increases the risk of systemic diseases (1). Consistently with this hypothesis, a number of studies have demonstrated the presence of periodontopathic bacterial DNA in various tissues and organs (4–6) and an elevated level of proinflammatory cytokines, in particular IL-6, in the blood (7–9). However, there is no direct evidence that the bacterial DNA detected is derived from bacteria that migrate from periodontal pockets into the body or that the cytokines detected in the blood are produced in the periodontal disease lesion.

Recently, we proposed a novel hypothesis regarding the link between periodontal disease and systemic diseases, based on the oral cavity-gut connection. We demonstrated that oral administration of P. gingivalis can change gut microbiota composition (dysbiosis), which is associated with impaired gut barrier function, resulting in endotoxemia and subsequent inflammation of the liver and adipose tissue (10, 11). Furthermore, P. gingivalis administration aggravates collagen-induced arthritis via gut dysbiosis and elevation of the Th17 response in the gut (12). These results strongly suggest that the gut dysbiosis and resulting endotoxemia and shift in the gut immune system toward a Th17-dominated response that are induced by ingesting periodontopathic bacteria might be the mechanism behind the association between periodontal and systemic diseases.

Several reports have suggested that a large part of the effect of gut dysbiosis on cardio-metabolic traits is attributable to microbial metabolites. It has been shown that an increased serum level of branched-chain amino acids (BCAA) is a characteristic feature of insulin resistance (13). Large-cohort studies have also demonstrated that high serum levels of BCAA and aromatic amino acids are significantly associated with future onset of diabetes (14) and cardiovascular diseases (15). More recently, a link between alterations in intestinal microbiota, circulating amino acids, and obesity has been reported (16). However, the relationship between P. gingivalis-induced alteration of gut microbiota and changes in metabolism has not yet been explored.

In this study, we investigated the effect of P. gingivalis administration on the serum levels of amino acids that are reported to be associated with various cardio-metabolic traits. We applied a bioinformatics approach to metabolomics to predict metagenome functional content from marker gene surveys.

## RESULTS

### Oral administration of P. gingivalis alters gut microbiota composition.

**FIG 1:**
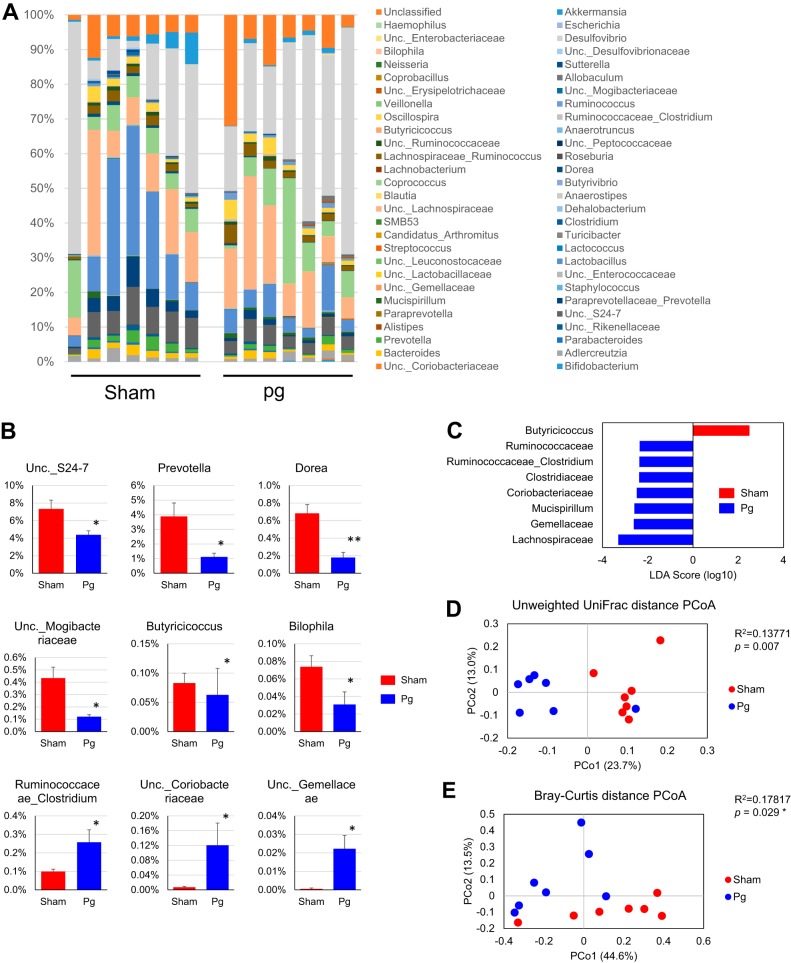
Effect of oral administration of P. gingivalis on gut microbiota composition. Male C57BL/6 mice (7/group) were orally administered with either 10^9^ CFU of live P. gingivalis or vehicle twice a week for 5 weeks. Fecal samples were subjected to 16S rRNA sequencing. (A) Relative abundances of bacterial groups at the genus level in the P. gingivalis- and sham-administered groups. (B) Bar plots for genera that exhibited significant differences in relative abundances between the groups. *, *P* < 0.05; **, *P* < 0.01, Mann-Whitney U test. (C) Linear discriminant analysis (LDA) scores derived from the LEfSe analysis. (D) PCoA of fecal microbiota from the two groups of mice using unweighted UniFrac distances (ANOSIM test). (E) PCoA of fecal microbiota from the two groups of mice using Bray*-*Curtis distances (ANOSIM test). Pg, P. gingivalis-administered mice; Sham, sham-administered mice; Unc., unclassified.

To assess the impact of oral administration of P. gingivalis on gut microbiota composition, we analyzed the composition, abundance, and function of gut microbiota in fecal samples, via high-throughput sequencing of the V4 regions of the 16S rRNA genes. We have previously confirmed that P. gingivalis can be detected in the oral cavity during the experimental period by the administration procedure used in this study (17). Since the number of high-quality reads varied from sample to sample, the read number obtained was rarefied by resampling (10,000 iterations) to the minimum sample size of all the groups that were compared. Microbial composition was analyzed to three taxonomic levels (Fig. 1A and see Fig. S1A in the supplemental material). At the phylum level, the proportion of *Bacteroidetes* was significantly lower in P. gingivalis-administered mice than in sham-administered mice, but the proportion of *Deferribacteres* was significantly higher (Fig. S1A). At the family level, the proportions of S24-7, *Paraprevotellaceae*, and *Mogibacteriaceae* were significantly lower in P. gingivalis-administered mice, but those of *Deferribacteriaceae*, *Gemellaceae*, and *Clostridiaceae* were significantly elevated (Fig. S1B).

10.1128/mSphere.00460-18.1 FIG S1 Effect of oral administration of P. gingivalis on gut microbiota composition. Male C57BL/6 mice (7/group) were orally administered with either 10^9^ CFUs of live P. gingivalis or vehicle twice a week for 5 weeks. Fecal samples were subjected to 16S rRNA sequencing. (A) Relative abundances of bacterial groups at the phylum and family levels in the P. gingivalis- and sham-administered groups. (B) Dot plots for phyla and families that exhibited significant differences in relative abundance between the groups. *, *P < *0.05; **, *P < *0.01, Mann-Whitney U test. Pg, P. gingivalis-administered mice; Sham, sham-administered mice; Unc., unclassified. Download FIG S1, PDF file, 0.1 MB. Copyright © 2018 Kato et al. 2018 Kato et al. This content is distributed under the terms of the Creative Commons Attribution 4.0 International license.

At the genus level, the proportions of unclassified *Coriobacteriaceae*, *Gemellaceae*, and *Clostridiaceae* were significantly elevated, while those of unclassified S24-7, *Prevotellaceae*, *Mogibacteriaceae*, *Dorea*, *Butyricicoccus*, and *Bilophila* were significantly lower in P. gingivalis-administered mice than in sham-administered mice (Fig. 1A and B). Biomarker analysis using linear discriminant analysis effect size (LEfSe) indicated that the P. gingivalis-administered mice were characterized by the family *Clostridiaceae*, phylum *Deferribacteres*, family *Gemellaceae*, and others. In contrast, the genus *Butyricicoccus* was characteristic in the sham-administered mice (Fig. 1C). Although the proportions of the genera *Lactobacillus* and *Desulfovibrio* seemed to be affected by P. gingivalis administration, the differences between the two groups of mice were not statistically significant. This shows that relatively minor populations of mouse gut microbiota were affected by P. gingivalis administration. In support of this idea, while a principal-component analysis (PCoA) of unweighted UniFrac distances (Fig. 1E) and a nonmetric multidimensional scaling plot (on a Bray-Curtis distance matrix) (Fig. 1F) revealed significant qualitative differences in gut microbiota compositions between the two groups, there was no difference in weighted UniFrac distances (data not shown).

### Effect of P. gingivalis administration on metabolic pathways.

**FIG 2:**
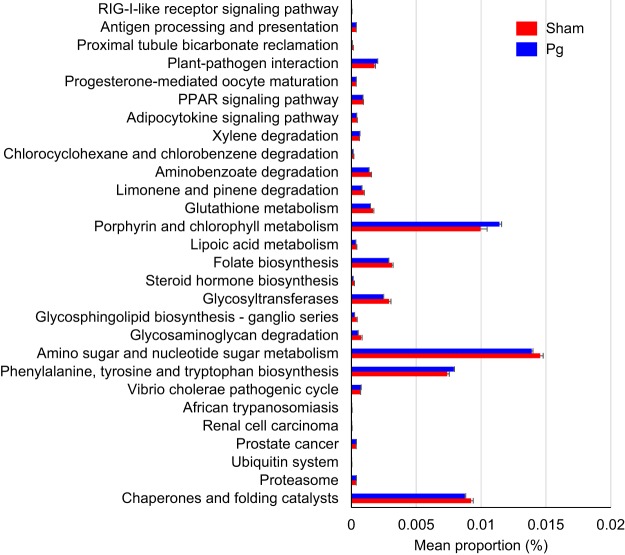
Differential PICRUSt-predicted KEGG pathways between P. gingivalis- and sham-administered mice, detected using HUMAnN2 software. The pathways demonstrating significant differences between two groups are shown. For each comparison, the mean proportion of predicted KEGG pathways (left) and differences in the proportions (mean ± standard deviations) (right) are illustrated. The NSTI (weighted nearest sequenced taxon index) scores for all samples were between 0.08 and 0.13. Pg, P. gingivalis-administered mice; Sham, sham-administered mice.

We used PICRUSt (phylogenetic investigation of communities by reconstruction of unobserved states) to infer metagenome functional content based on the microbial community profiles obtained from the 16S rRNA gene sequences. Figure 2 depicts the general metabolic pathways and compares microbiota functions between the two groups of mice, highlighting the significant differences in the distribution of metabolic pathways. Significant differences were observed in pathways relating to amino acid metabolism: phenylalanine, tyrosine, and tryptophan biosynthesis and porphyrin and chlorophyll metabolism. Overall, the microbial communities present in the two groups could be distinguished based on their functions. However, the predicted KEGG (Kyoto Encyclopedia of Genes and Genomes) pathways that were significantly reduced in P. gingivalis-administered mice were amino sugar and nucleotide sugar metabolism, chaperones and folding catalysts, glucosyltransferases, limonene and pinene degradation, and folate biosynthesis.

**FIG 3:**
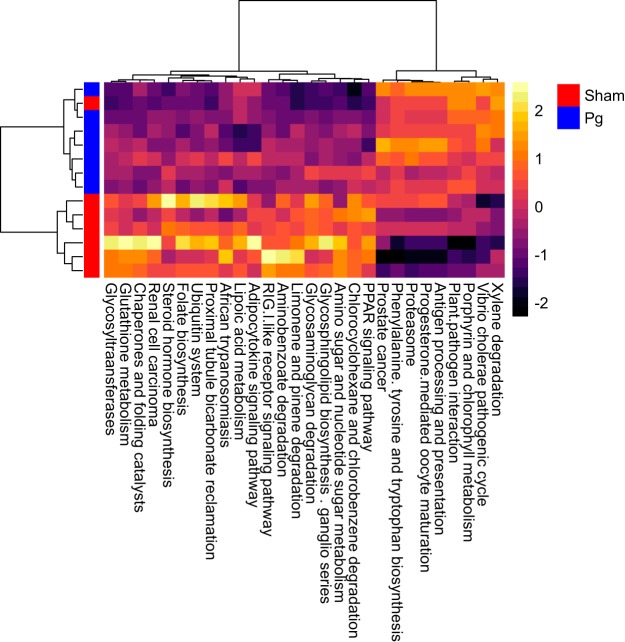
Heat map and hierarchical clustering of reporter scores for KEGG pathways in P. gingivalis*-* and sham-administered mice. The patterns for some pathways differ between the two groups. Pg, P. gingivalis-administered mice; Sham, sham-administered mice.

**FIG 4:**
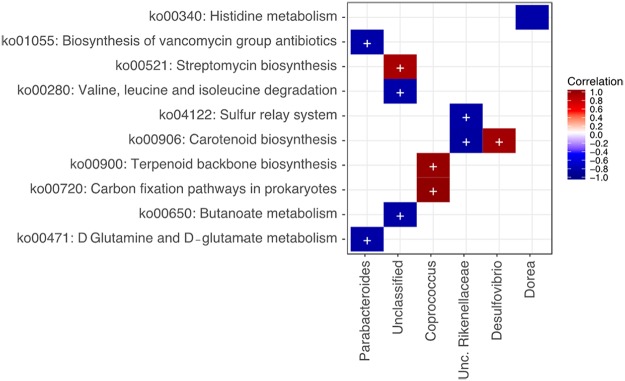
Association between gut microbiota at the genus level and KEGG pathways. Data were analyzed using HUMAnN2 software. Significant associations are indicated using colored blocks.

These differences between the two groups were also revealed by hierarchical clustering using the intensity of each metabolic pathway that was detected in each sample (Fig. 3). With respect to correlations between KEGG Orthology information and microbiota composition at the genus level, there was a significant positive correlation between *Coprococcus* and both terpenoid backbone biosynthesis and carbon fixation and between *Desulfovibrio* and carotenoid biosynthesis (Fig. 4). There was a significant negative correlation between *Parabacteroides* and both vancomycin group antibiotic biosynthesis and d-glutamine and -glutamate metabolism and between *Rikenellaceae* and both the sulfur relay system and carotenoid biosynthesis. Unclassified genera were significantly positively correlated with streptomycin biosynthesis and negatively correlated with both valine, leucine, and isoleucine degradation and butanoate metabolism.

### Serum metabolic profiling of P. gingivalis-administered mice.

**FIG 5:**
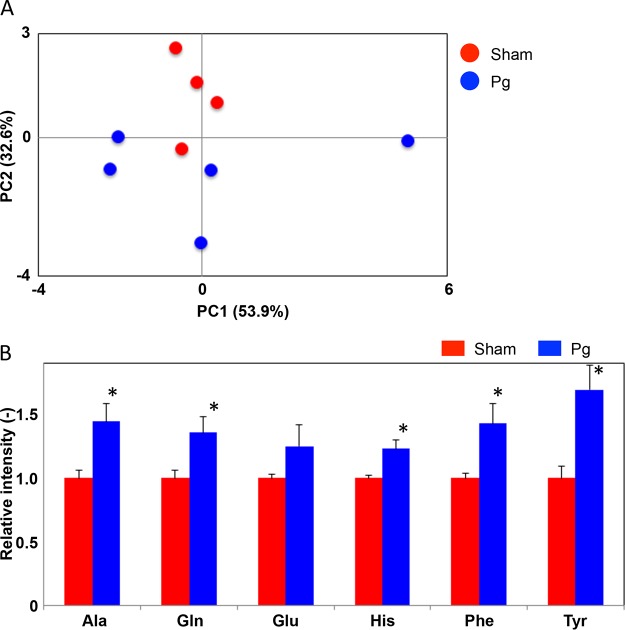
Effect of oral administration of P. gingivalis on serum metabolites. (A) PCA of serum metabolites from P. gingivalis- and sham-administered mice. (B) Serum amino acids that differed in abundance between the two groups. Alanine (Ala), glutamine (Gln), histidine (His), tyrosine (Tyr), and phenylalanine (Phe) were significantly increased in the P. gingivalis-administered mice, and glutamate (Glu) was nonsignificantly increased. *, *P* < 0.05, Mann-Whitney U test. Pg, P. gingivalis-administered mice; Sham, sham-administered mice.

We further analyzed some of the serum samples to assess whether P. gingivalis administration could perturb the murine metabolome. We performed an untargeted analysis of all the data acquired to examine a wider pool of metabolites. A PCA was conducted using nuclear magnetic resonance (NMR)-derived data to provide an overview of the differences between P. gingivalis- and sham-administered mice. This confirmed the existence of differences associated with P. gingivalis administration, as reflected by differential clustering of the two groups (Fig. 5A). As we predicted and inferred from our PICRUSt analysis, serum metabolite profiles were affected along with the gut microbiota. Of the annotated signals, alanine, glutamine, histidine, tyrosine, and phenylalanine were significantly higher in the P. gingivalis-administered mice (Fig. 5B).

## DISCUSSION

In the present study, we demonstrated that oral administration of P. gingivalis alters the serum metabolite profile and confirmed the change in gut microbiota composition in mice. The PICRUSt analysis revealed a number of increased and decreased KEGG pathways. Among the increased KEGG orthologs, the phenylalanine, tyrosine, and tryptophan biosynthesis pathways were of particular interest. Consistently with these *in silico* analyses, serum metabolome analysis uncovered a significant increase in phenylalanine and tyrosine in P. gingivalis-administered mice compared with that in sham-administered mice. These findings suggest that oral administration of P. gingivalis affects gut microbiota composition such that it is biased toward an increased production of these amino acids and that elevated levels thereof are absorbed into systemic circulation.

Periodontal disease is not just a chronic inflammatory disease affecting the periodontium and leading to tooth loss if left untreated, but it is also believed to increase the risk of various systemic diseases, such as atherosclerotic vascular diseases and type 2 diabetes. However, the exact mechanisms by which periodontal disease adversely affects systemic health have not yet been elucidated. We recently proposed the oral-cavity–gut–multiorgan axis as a conceptual idea for the connection between periodontal disease and systemic diseases. This is based on findings that orally administered P. gingivalis alters gut microbiota composition, reduces gut barrier function, and modulates the gut immune system, resulting in an increased systemic inflammatory state. In addition, oral administration of P. gingivalis induces a change in gut immune profile toward Th17 dominance, concomitant with alterations in gut microbiota in collagen-induced-arthritis models. However, the underlying mechanisms by which gut dysbiosis induced by oral administration of P. gingivalis results in these pathological changes have not been fully elucidated.

There are several mechanisms by which gut dysbiosis causes the deterioration of systemic physiological functions. These include inducing endotoxemia via the impairment of gut barrier function (18), modulating the gut immune system (19), and altering the relative quantities of various bacterial metabolites (20). The gut microbiota are exclusively responsible for several important metabolic functions, including vitamin and short-chain fatty acid production, amino acid synthesis, bile acid biotransformation, hydrolysis, and the fermentation of nondigestible substrates (21). By these actions, the gut microbiota modulates signaling pathways involved in homeostasis. Thus, when balanced interaction between the gastrointestinal tract and the resident microbiota is disrupted, intestinal and extraintestinal diseases may develop or deteriorate. Allergies, obesity, cancer, diabetes, metabolic disorders, cardiovascular disease, neuropathology, and even periodontitis can result from this disruption.

Recently, serum levels of metabolites, especially those of amino acids, were shown to be associated with metabolic diseases. It was reported that branched-chain amino acids (BCAA) contribute to the development of obesity-associated insulin resistance (22). Furthermore, a prospective cohort study demonstrated that the level of serum BCAA can predict the future development of diabetes (14) and cardiovascular disease (15). The same cohort study also revealed an association between BCAA and body mass index (BMI) and showed that BMI-related metabolites were connected to some gut microbiota genera (23). In addition to BCAA, aromatic amino acids (phenylalanine, tryptophan, tyrosine) have been shown to be associated with the risk of developing diabetes (14) and cardiovascular disease (15).

Although the underlying mechanisms by which serum amino acids increase the risk of developing metabolic diseases have not been fully elucidated, one mechanism has been proposed that might explain how elevated levels of BCAA might be linked to metabolic diseases. Increased plasma BCAA levels stimulate the activation of mammalian target of rapamycin complex 1 (mTORC1) and serine kinases S6K1 in skeletal muscles. Persistent activation of mTORC1 and S6K1 induce serine phosphorylation of insulin receptor substrate 1 (IRS-1) and IRS-2. These mechanisms have been implicated in diet-induced insulin resistance (22). However, numerous observations indicate that BCAA-associated mTORC1 activation is not necessary or sufficient to trigger insulin resistance (24).

Increased levels of serum aromatic amino acids are also reported to be associated with a risk of developing diabetes (14) and obesity (16). These findings further support the implication of the role of periodontal disease in the development of diabetes and obesity-related diseases via the gut microbiota (10). However, the mechanisms linking elevated levels of aromatic amino acids and impaired metabolic status are still unknown.

Another noteworthy result is that the amino acid glutamate tended to be elevated in P. gingivalis-administered mice relative to levels in sham-administered mice. As with BCAA and aromatic amino acids, glutamate has been reported to be associated with obesity (23). Although no association between serum glutamate levels and the severity of periodontitis or disease types has yet been reported, given the relationship between periodontitis and obesity or metabolic diseases, clinical studies are warranted. Ottosson et al. showed that the obesity-related metabolite glutamate is positively associated with three genera, *Blautia*, *Dorea*, and *Ruminococcus*, all of which are members of the family *Lachnospiraceae* (23).

In the present study, the proportion of *Ruminococcus* tended to be higher in P. gingivalis-administered mice than in sham-administered mice. In contrast, the proportion of *Dorea* species was significantly lower in P. gingivalis-administered mice, and there was no difference in the proportions of *Blautia* species. However, we were unable to clarify the causal relationship between the *Lachnospiraceae* and glutamate levels in mice, and 16S rRNA gene sequencing also does not allow us to study bacteria at the species level. Nevertheless, several positive and negative associations between KEGG pathways and bacterial genera were found. Further studies are needed to identify the mechanisms behind the pathological changes that are induced by oral administration of P. gingivalis or the dysbiosis of oral microbiota.

## MATERIALS AND METHODS

### Ethics statement.

This study was approved by the Institutional Animal Care and Use Committee at Niigata University (permit number 227-5). All experiments were performed in accordance with the Regulations and Guidelines on Scientific and Ethical Care and Use of Laboratory Animals of the Science Council of Japan, enforced on 1 June 2006.

### Infection of mice with P. gingivalis.

Six-week-old male C57BL/6 mice were obtained from Japan SLC (Shizuoka, Japan). The mice were acclimatized under specific*-*pathogen-free conditions and fed regular chow and sterile water until the commencement of infection at 7 weeks of age. The mice were orally administered with either live P. gingivalis strain W83 (10^9^ CFU) or vehicle (phosphate-buffered saline [PBS] with 2% carboxymethyl cellulose) twice a week for 5 weeks (7 mice/group). The details of the bacterial culture and the procedures for oral administration have been described previously (10–12).

### DNA extraction from fecal samples.

DNA was extracted from feces as described previously (25). In brief, feces were collected 24 h after the final administration of P. gingivalis and then freeze-dried. Prior to 16S rRNA gene sequencing, the freeze-dried feces were suspended in buffer containing 10% sodium dodecyl sulfate and 10 mM Tris-HCl, 1 mM EDTA (pH 8.0). Feces suspended in the buffer were then disrupted with 0.1-mm zirconia/silica beads (BioSpec Products, Bartlesville, OK, USA) by shaking (1,500 rpm, 10 min) using a ShakeMaster (Hirata, Tokyo, Japan). After centrifugation, bacterial DNA was purified using a 25:24:1 phenol-chloroform-isoamyl alcohol solution. The DNA was precipitated by adding ethanol and sodium acetate. RNase treatment and polyethylene glycol precipitation were also performed.

### Gut and liver microbiota analysis via 16S rRNA sequencing.

The V4 variable region (515F to 806R) of the respective samples was sequenced using Illumina MiSeq by following the method of Kozich et al. (26). Each reaction mixture contained 15 pmol of each primer, 0.2 mM deoxyribonucleoside triphosphates, 5 μl of 10× *Ex Taq* HS buffer, 1.25 U *Ex Taq* HS polymerase (TaKaRa Bio, Shiga, Japan), 50 ng extracted DNA, and sterilized water, to achieve a final volume of 50 μl. The PCR profile was set as follows: 95°C, 2 min, 25 cycles of 95°C for 20 s, 55°C for 15 s, and 72°C for 5 min, followed by 72°C for 10 min.

The PCR products were purified using AMPure XP (Beckman Coulter, Brea, CA, USA) and quantified using a Quant-iT PicoGreen double-stranded DNA (dsDNA) assay kit (Life Technologies Japan, Tokyo, Japan). Mixed samples were prepared by pooling approximately equal amounts of PCR amplicons from each sample. The pooled library was analyzed using an Agilent high-sensitivity DNA kit on an Agilent 2100 bioanalyzer. Real-time PCR quantification was performed on the pooled library using the KAPA library quantification kit for Illumina by following the manufacturer’s protocols.

Based on the quantification, the sample library was denatured and diluted. A sample library containing 20% denatured PhiX spike-in was sequenced via MiSeq using a 500-cycle kit. We obtained 2× 250-bp paired-end reads.

Taxonomic assignments and the estimation of relative abundances from sequencing data were performed using the analysis pipeline of the QIIME software package (27). An operational taxonomic unit (OTU) was defined at 97% similarity. OTUs indicating relative abundances of under 0.1% were filtered to remove noise. OTU taxonomy was assigned based on comparison with the Silva database, using UCLUST (28, 29). Beta diversity was calculated using the Bray-Curtis distance metric and visualized by principal-coordinate analysis (PCoA) using weighted or unweighted UniFrac distances based on the OTU distribution across samples (30).

PICRUSt (phylogenetic investigation of communities by reconstruction of unobserved states) (31) was then applied to the OTU table selected by Greengenes (the Greengenes 16S rRNA gene database and tools) to generate metagenomic data and derive KEGG Orthology gene abundance data. KEGG Orthology gene family abundances were summarized at a higher hierarchical level in pathway-level categories for easier biological interpretation. Nonmicrobial categories, such as organismal systems and human diseases were excluded from further analysis. The associated metabolic pathways were deciphered by employing HUMAnN2 (The HMP Unified Metabolic Analysis Network) with the default settings. To characterize the accuracy of PICRUSt, the weighted nearest sequenced taxon index (weighted NSTI) was calculated. Samples with a weighted NSTI of <0.15 were excluded from the analysis.

### Measurements of serum metabolites.

Serum samples from P. gingivalis-administered (*n* = 5) and sham-administered (*n* = 4) mice diluted to one-sixth of their original concentration in 100 mmol/liter potassium phosphate buffer (in deuterium oxide containing 1 mmol/liter sodium 2,2-dimethyl-2-silapentane-5-sulfonate, pH 7.0) were measured on an NMR spectrometer (Bruker Avance II 700; Bruker Biospin, Rheinstetten, Germany) as described previously (32). Briefly, all ^1^H NMR spectra were acquired using a Bruker standard-pulse program, cpmgpr1d, with 32,000 data points, 32 scans, 16 dummy scans, a 16-ppm spectral width, and a 3-s relaxation delay. For annotation of the signals detected in ^1^H NMR spectra, two-dimensional *J*-resolved NMR measurements were performed using a Bruker standard-pulse program, jresgpprqf, with 32 data points for F1 and 16,000 data points for F2, 8 scans, 16 dummy scans, a 50-Hz spectral width for F1, a 16-ppm spectral width for F2, and a 1.5-s relaxation delay as described previously (33). The detected signals were annotated using the SpinCouple program (34) (http://dmar.riken.jp/spincouple/) with reference to the Human Metabolome Database (35) (http://www.hmdb.ca/).

### Statistical analyses.

Nonparametric data were evaluated using the Mann-Whitney U test for two-group comparisons in GraphPad Prism (GraphPad Software, La Jolla, CA, USA). Analysis of similarity (ANOSIM) was performed to test differences in bacterial community compositions between the groups (P. gingivalis- and sham-administered samples) using the vegan package in R (http://cran.at.r-project.org/). This package calculates *R* as a statistical value that describes the level of similarity between each pair in ANOSIM. Values close to unity indicate completely different communities, whereas a zero value indicates a complete overlap or similarity (null hypothesis). A *P* value of <0.05 was considered statistically significant.

### Data availability.

All relevant data have been included in the article. We will provide any additional data upon request.

## References

[1] Yamazaki K 2016 New paradigm in the relationship between periodontal disease, and systemic diseases: effects of oral bacteria on the gut microbiota and metabolism. p 243–261. *In* Nibali L, Henderson B (ed), The human microbiota and chronic disease: dysbiosis as cause of human pathology, 1st ed. John Wiley & Sons, Inc, Oxford, United Kingdom.

[2] Lindhe J, Liljenberg B, Listgarten M 1980 Some microbiological and histopathological features of periodontal disease in man. J Periodontol 51:264–269. doi:10.1902/jop.1980.51.5.264. 6929912

[3] Gemmell E, Yamazaki K, Seymour GJ 2007 The role of T cells in periodontal disease: homeostasis and autoimmunity. Periodontol 2000 43:14–40. doi:10.1111/j.1600-0757.2006.00173.x. 17214833

[4] Kozarov EV, Dorn BR, Shelburne CE, Dunn WA Jr, Progulske-Fox A 2005 Human atherosclerotic plaque contains viable invasive *Actinobacillus actinomycetemcomitans* and *Porphyromonas gingivalis*. Arterioscler Thromb Vasc Biol 25:e17–e18. doi:10.1161/01.ATV.0000155018.67835.1a. 15662025

[5] Rafferty B, Jonsson D, Kalachikov S, Demmer RT, Nowygrod R, Elkind MS, Bush H Jr, Kozarov E 2011 Impact of monocytic cells on recovery of uncultivable bacteria from atherosclerotic lesions. J Intern Med 270:273–280. doi:10.1111/j.1365-2796.2011.02373.x. 21366733 PMC3133811

[6] Tomas I, Diz P, Tobias A, Scully C, Donos N 2012 Periodontal health status and bacteraemia from daily oral activities: systematic review/meta-analysis. J Clin Periodontol 39:213–228. doi:10.1111/j.1600-051X.2011.01784.x. 22092606

[7] Nakajima T, Honda T, Domon H, Okui T, Kajita K, Ito H, Takahashi N, Maekawa T, Tabeta K, Yamazaki K 2010 Periodontitis-associated up-regulation of systemic inflammatory mediator level may increase the risk of coronary heart disease. J Periodontal Res 45:116–122. doi:10.1111/j.1600-0765.2009.01209.x. 19602107

[8] Schenkein HA, Loos BG 2013 Inflammatory mechanisms linking periodontal diseases to cardiovascular diseases. J Clin Periodontol 40(Suppl 14):S51–S69. doi:10.1111/jcpe.12060. 23627334 PMC4554326

[9] Vidal F, Figueredo CM, Cordovil I, Fischer RG 2009 Periodontal therapy reduces plasma levels of interleukin-6, C-reactive protein, and fibrinogen in patients with severe periodontitis and refractory arterial hypertension. J Periodontol 80:786–791. doi:10.1902/jop.2009.080471. 19405832

[10] Arimatsu K, Yamada H, Miyazawa H, Minagawa T, Nakajima M, Ryder MI, Gotoh K, Motooka D, Nakamura S, Iida T, Yamazaki K 2014 Oral pathobiont induces systemic inflammation and metabolic changes associated with alteration of gut microbiota. Sci Rep 4:4828. doi:10.1038/srep04828. 24797416 PMC4010932

[11] Nakajima M, Arimatsu K, Kato T, Matsuda Y, Minagawa T, Takahashi N, Ohno H, Yamazaki K 2015 Oral administration of *P. gingivalis* induces dysbiosis of gut microbiota and impaired barrier function leading to dissemination of enterobacteria to the liver. PLoS One 10:e0134234. doi:10.1371/journal.pone.0134234. 26218067 PMC4517782

[12] Sato K, Takahashi N, Kato T, Matsuda Y, Yokoji M, Yamada M, Nakajima T, Kondo N, Endo N, Yamamoto R, Noiri Y, Ohno H, Yamazaki K 2017 Aggravation of collagen-induced arthritis by orally administered *Porphyromonas gingivalis* through modulation of the gut microbiota and gut immune system. Sci Rep 7:6955. doi:10.1038/s41598-017-07196-7. 28761156 PMC5537233

[13] Pedersen HK, Gudmundsdottir V, Nielsen HB, Hyotylainen T, Nielsen T, Jensen BA, Forslund K, Hildebrand F, Prifti E, Falony G, Le Chatelier E, Levenez F, Dore J, Mattila I, Plichta DR, Poho P, Hellgren LI, Arumugam M, Sunagawa S, Vieira-Silva S, Jorgensen T, Holm JB, Trost K, Kristiansen K, Brix S, Raes J, Wang J, Hansen T, Bork P, Brunak S, Oresic M, Ehrlich SD, Pedersen O 2016 Human gut microbes impact host serum metabolome and insulin sensitivity. Nature 535:376–381. doi:10.1038/nature18646. 27409811

[14] Wang TJ, Larson MG, Vasan RS, Cheng S, Rhee EP, McCabe E, Lewis GD, Fox CS, Jacques PF, Fernandez C, O’Donnell CJ, Carr SA, Mootha VK, Florez JC, Souza A, Melander O, Clish CB, Gerszten RE 2011 Metabolite profiles and the risk of developing diabetes. Nat Med 17:448–453. doi:10.1038/nm.2307. 21423183 PMC3126616

[15] Magnusson M, Lewis GD, Ericson U, Orho-Melander M, Hedblad B, Engstrom G, Ostling G, Clish C, Wang TJ, Gerszten RE, Melander O 2013 A diabetes-predictive amino acid score and future cardiovascular disease. Eur Heart J 34:1982–1989. doi:10.1093/eurheartj/ehs424. 23242195 PMC3703309

[16] Liu R, Hong J, Xu X, Feng Q, Zhang D, Gu Y, Shi J, Zhao S, Liu W, Wang X, Xia H, Liu Z, Cui B, Liang P, Xi L, Jin J, Ying X, Wang X, Zhao X, Li W, Jia H, Lan Z, Li F, Wang R, Sun Y, Yang M, Shen Y, Jie Z, Li J, Chen X, Zhong H, Xie H, Zhang Y, Gu W, Deng X, Shen B, Xu X, Yang H, Xu G, Bi Y, Lai S, Wang J, Qi L, Madsen L, Wang J, Ning G, Kristiansen K, Wang W 2017 Gut microbiome and serum metabolome alterations in obesity and after weight-loss intervention. Nat Med 23:859–868. doi:10.1038/nm.4358. 28628112

[17] Maekawa T, Takahashi N, Tabeta K, Aoki Y, Miyashita H, Miyauchi S, Miyazawa H, Nakajima T, Yamazaki K 2011 Chronic oral infection with *Porphyromonas gingivalis* accelerates atheroma formation by shifting the lipid profile. PLoS One 6:e20240. doi:10.1371/journal.pone.0020240. 21625524 PMC3098290

[18] Cani PD 2012 Crosstalk between the gut microbiota and the endocannabinoid system: impact on the gut barrier function and the adipose tissue. Clin Microbiol Infect 18(Suppl 4):50–53. doi:10.1111/j.1469-0691.2012.03866.x. 22647050

[19] Luo A, Leach ST, Barres R, Hesson LB, Grimm MC, Simar D 2017 The microbiota and epigenetic regulation of T helper 17/regulatory T cells: in search of a balanced immune system. Front Immunol 8:417. doi:10.3389/fimmu.2017.00417. 28443096 PMC5385369

[20] Sharon G, Garg N, Debelius J, Knight R, Dorrestein PC, Mazmanian SK 2014 Specialized metabolites from the microbiome in health and disease. Cell Metab 20:719–730. doi:10.1016/j.cmet.2014.10.016. 25440054 PMC4337795

[21] Putignani L, Del Chierico F, Vernocchi P, Cicala M, Cucchiara S, Dallapiccola B 2016 Gut microbiota dysbiosis as risk and premorbid factors of IBD and IBS along the childhood-adulthood transition. Inflamm Bowel Dis 22:487–504. doi:10.1097/MIB.0000000000000602. 26588090

[22] Newgard CB, An J, Bain JR, Muehlbauer MJ, Stevens RD, Lien LF, Haqq AM, Shah SH, Arlotto M, Slentz CA, Rochon J, Gallup D, Ilkayeva O, Wenner BR, Yancy WS Jr, Eisenson H, Musante G, Surwit RS, Millington DS, Butler MD, Svetkey LP 2009 A branched-chain amino acid-related metabolic signature that differentiates obese and lean humans and contributes to insulin resistance. Cell Metab 9:311–326. doi:10.1016/j.cmet.2009.02.002. 19356713 PMC3640280

[23] Ottosson F, Brunkwall L, Ericson U, Nilsson PM, Almgren P, Fernandez C, Melander O, Orho-Melander M 2018 Connection between BMI related plasma metabolite profile and gut microbiota. J Clin Endocrinol Metab 103:1491–1501. doi:10.1210/jc.2017-02114. 29409054

[24] Lynch CJ, Adams SH 2014 Branched-chain amino acids in metabolic signalling and insulin resistance. Nat Rev Endocrinol 10:723–736. doi:10.1038/nrendo.2014.171. 25287287 PMC4424797

[25] Kato T, Fukuda S, Fujiwara A, Suda W, Hattori M, Kikuchi J, Ohno H 2014 Multiple omics uncovers host-gut microbial mutualism during prebiotic fructooligosaccharide supplementation. DNA Res 21:469–480. doi:10.1093/dnares/dsu013. 24848698 PMC4195493

[26] Kozich JJ, Westcott SL, Baxter NT, Highlander SK, Schloss PD 2013 Development of a dual-index sequencing strategy and curation pipeline for analyzing amplicon sequence data on the MiSeq Illumina sequencing platform. Appl Environ Microbiol 79:5112–5120. doi:10.1128/AEM.01043-13. 23793624 PMC3753973

[27] Caporaso JG, Kuczynski J, Stombaugh J, Bittinger K, Bushman FD, Costello EK, Fierer N, Peña AG, Goodrich JK, Gordon JI, Huttley GA, Kelley ST, Knights D, Koenig JE, Ley RE, Lozupone CA, McDonald D, Muegge BD, Pirrung M, Reeder J, Sevinsky JR, Turnbaugh PJ, Walters WA, Widmann J, Yatsunenko T, Zaneveld J, Knight R 2010 QIIME allows analysis of high-throughput community sequencing data. Nat Methods 7:335–336. doi:10.1038/nmeth.f.303. 20383131 PMC3156573

[28] Edgar RC 2010 Search and clustering orders of magnitude faster than BLAST. Bioinformatics 26:2460–2461. doi:10.1093/bioinformatics/btq461. 20709691

[29] Quast C, Pruesse E, Yilmaz P, Gerken J, Schweer T, Yarza P, Peplies J, Glockner FO 2013 The SILVA ribosomal RNA gene database project: improved data processing and web-based tools. Nucleic Acids Res 41:D590–D596. doi:10.1093/nar/gks1219. 23193283 PMC3531112

[30] Abdollahi-Roodsaz S, Joosten LA, Koenders MI, Devesa I, Roelofs MF, Radstake TR, Heuvelmans-Jacobs M, Akira S, Nicklin MJ, Ribeiro-Dias F, van den Berg WB 2008 Stimulation of TLR2 and TLR4 differentially skews the balance of T cells in a mouse model of arthritis. J Clin Invest 118:205–216. doi:10.1172/JCI32639. 18060042 PMC2104479

[31] Langille MG, Zaneveld J, Caporaso JG, McDonald D, Knights D, Reyes JA, Clemente JC, Burkepile DE, Vega Thurber RL, Knight R, Beiko RG, Huttenhower C 2013 Predictive functional profiling of microbial communities using 16S rRNA marker gene sequences. Nat Biotechnol 31:814–821. doi:10.1038/nbt.2676. 23975157 PMC3819121

[32] Motegi H, Tsuboi Y, Saga A, Kagami T, Inoue M, Toki H, Minowa O, Noda T, Kikuchi J 2015 Identification of reliable components in multivariate curve resolution-alternating least squares (MCR-ALS): a data-driven approach across metabolic processes. Sci Rep 5:15710. doi:10.1038/srep15710. 26531245 PMC4632111

[33] Misawa T, Wei F, Kikuchi J 2016 Application of two-dimensional nuclear magnetic resonance for signal enhancement by spectral integration using a large data set of metabolic mixtures. Anal Chem 88:6130–6134. doi:10.1021/acs.analchem.6b01495. 27257670

[34] Kikuchi J, Tsuboi Y, Komatsu K, Gomi M, Chikayama E, Date Y 2016 SpinCouple: development of a web tool for analyzing metabolite mixtures via two-dimensional J-resolved NMR database. Anal Chem 88:659–665. doi:10.1021/acs.analchem.5b02311. 26624790

[35] Wishart DS, Jewison T, Guo AC, Wilson M, Knox C, Liu Y, Djoumbou Y, Mandal R, Aziat F, Dong E, Bouatra S, Sinelnikov I, Arndt D, Xia J, Liu P, Yallou F, Bjorndahl T, Perez-Pineiro R, Eisner R, Allen F, Neveu V, Greiner R, Scalbert A 2013 HMDB 3.0*–*The Human Metabolome Database in 2013. Nucleic Acids Res 41:D801–D807. doi:10.1093/nar/gks1065. 23161693 PMC3531200

