# Supplementary figures and images for: Oral Administration of Porphyromonas gingivalis Alters the Gut Microbiome and Serum Metabolome
Source: mSphere. 2018 Oct 17;3(5):e00460-18. doi: 10.1128/mSphere.00460-18 (PMC6193602; doi:10.1128/mSphere.00460-18)

Supplementary Figure 1A

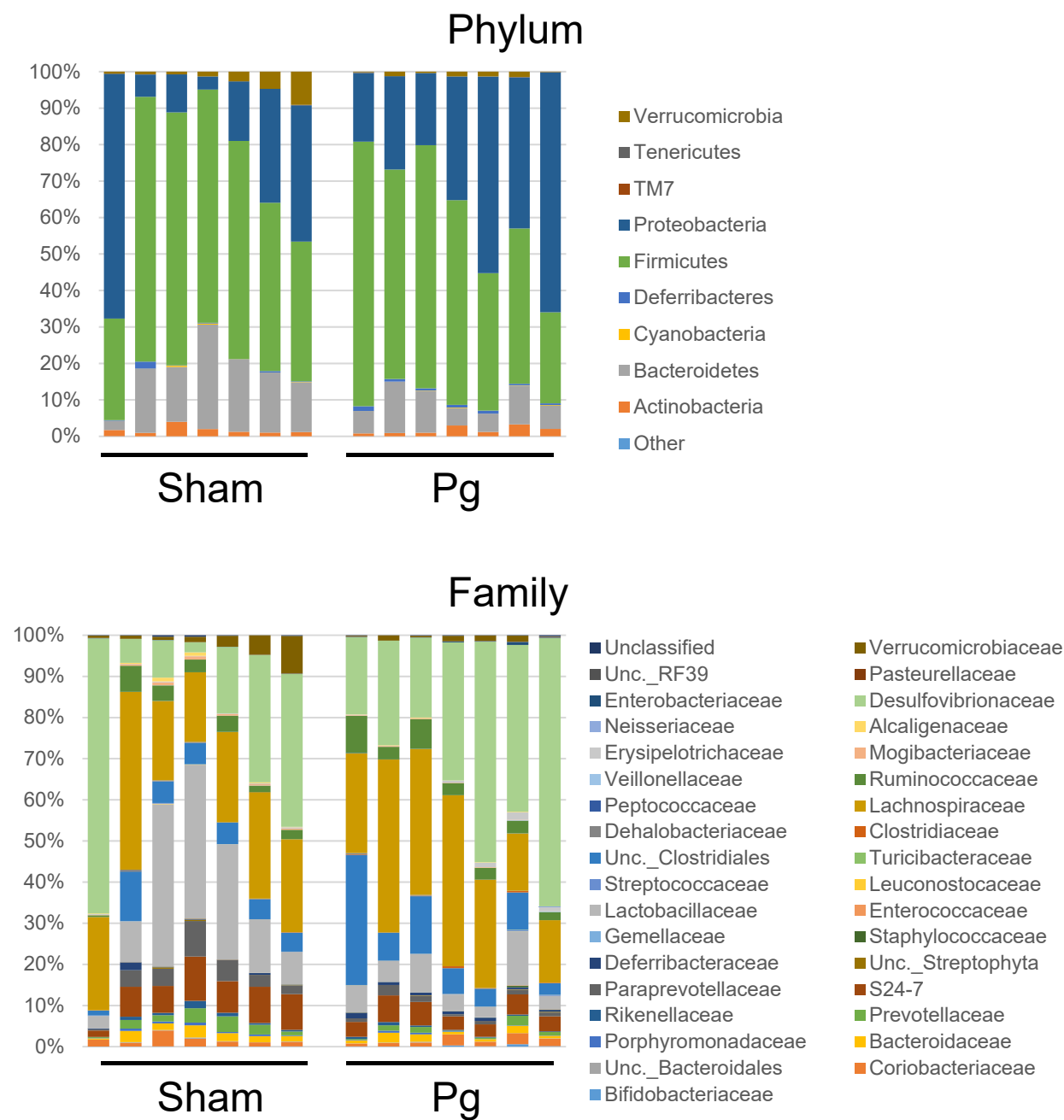

Supplementary Figure 1B

Phylum

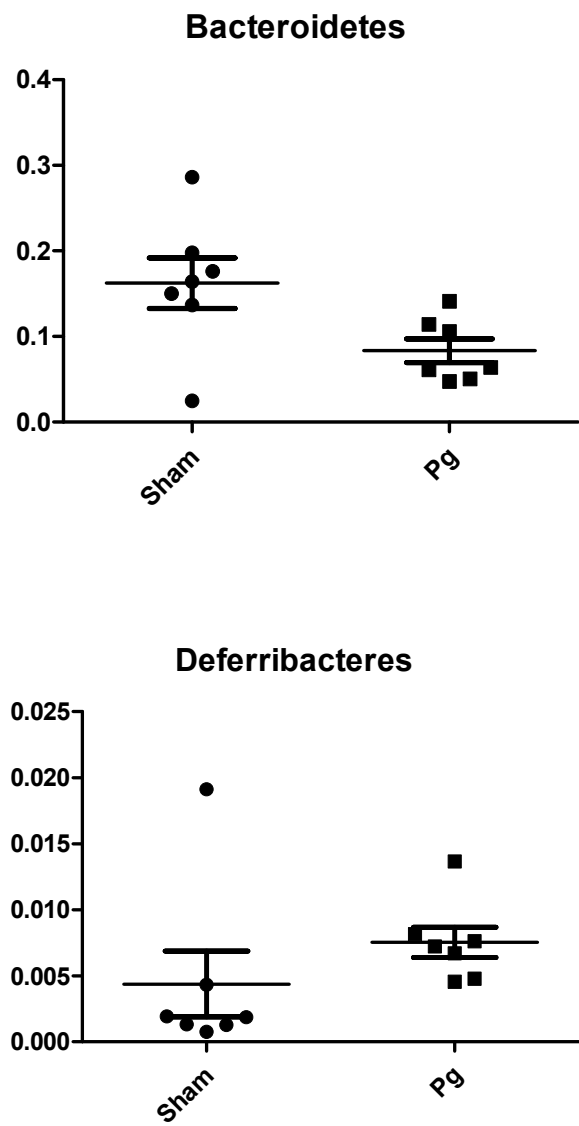

Family

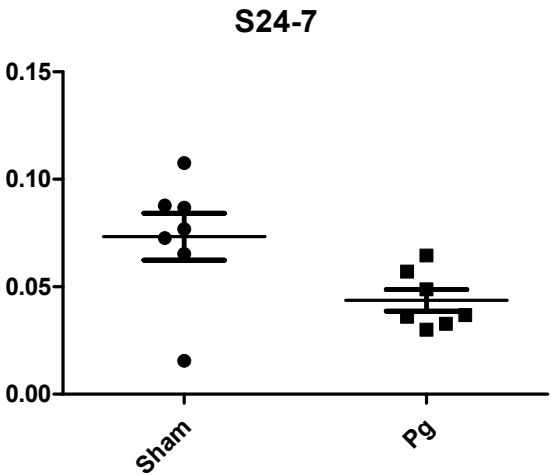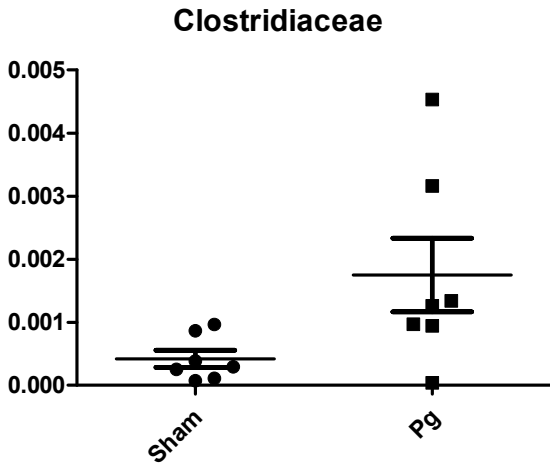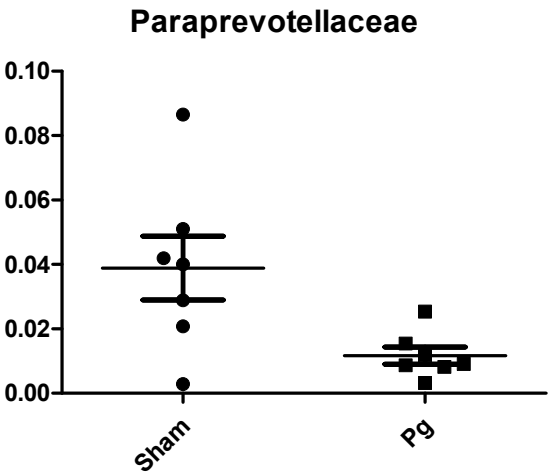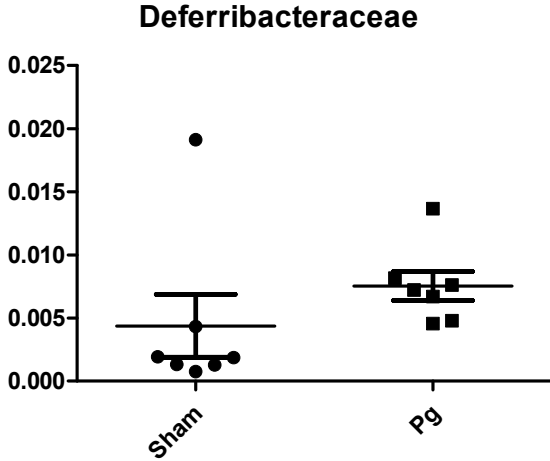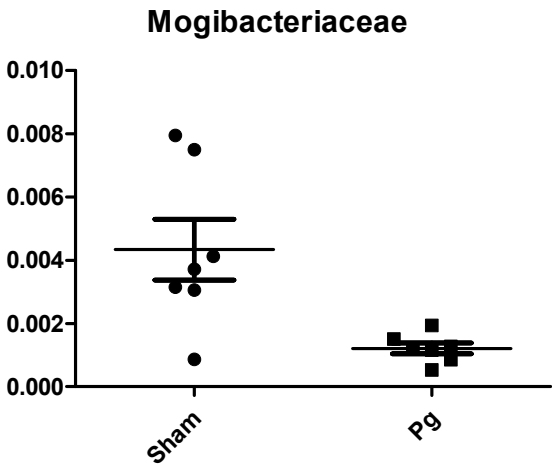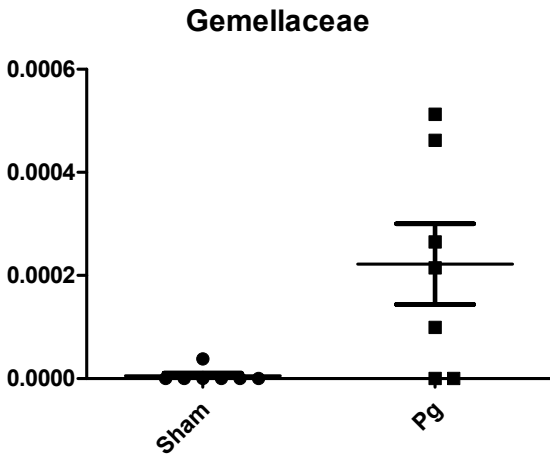

Figure 1B

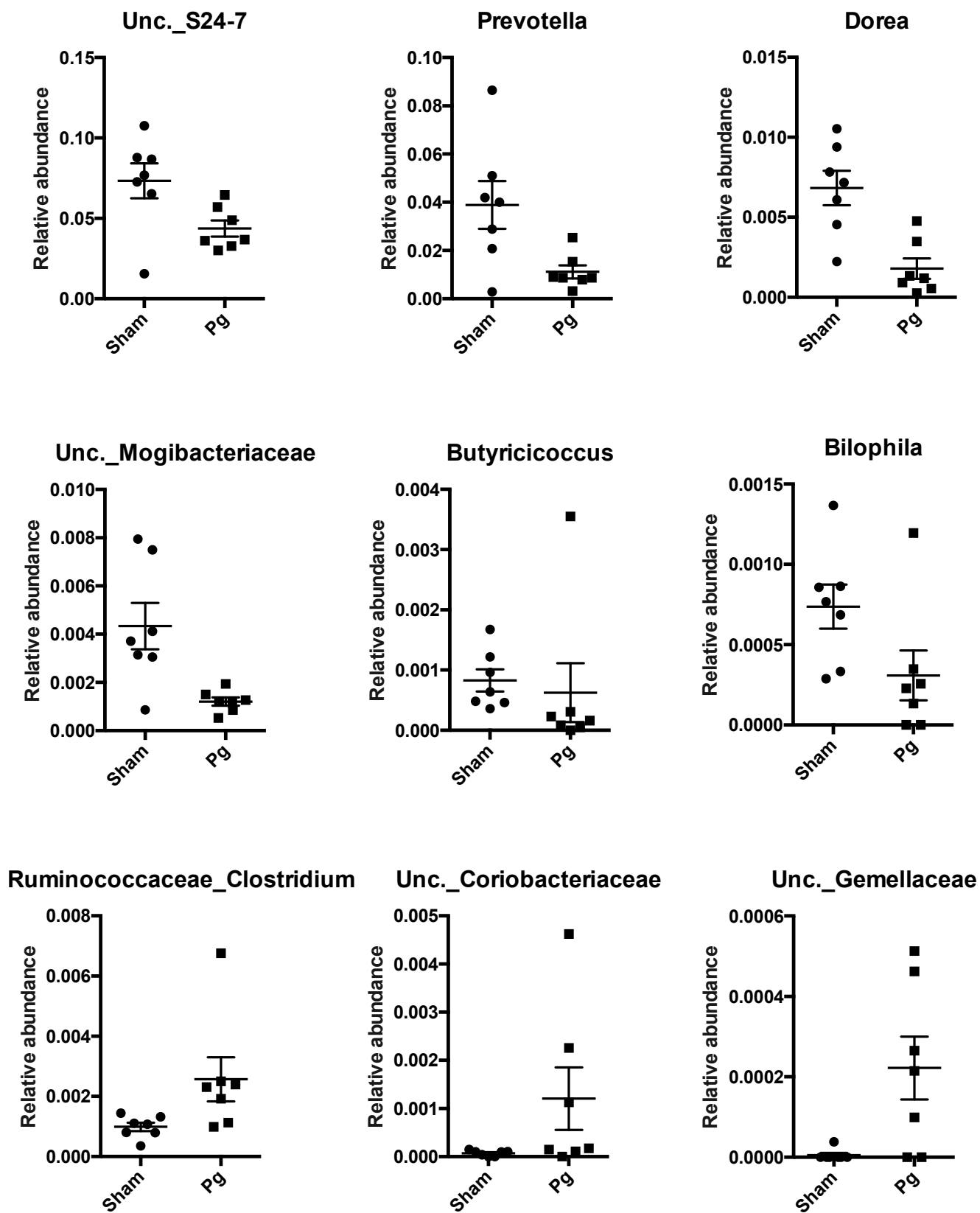

Supplement: FIG S1 [file sph005182660sf1.pdf]
